# Improving Clarity and Interpretability of Items in a Bilingual Index of Propensity to Integrate Research Evidence Into Clinical Decision‐Making in Rehabilitation

**DOI:** 10.1111/jep.70196

**Published:** 2025-07-09

**Authors:** Jacqueline Roberge‐Dao, Nancy Mayo, Annie Rochette, Keiko Shikako, Aliki Thomas

**Affiliations:** ^1^ School of Physical and Occupational Therapy McGill University Montréal Canada; ^2^ Centre for Interdisciplinary Research in Rehabilitation of Greater Montréal Montréal Canada; ^3^ Center for Outcomes Research and Evaluation (CORE) The Research Institute of the McGill University Health Center (RI‐MUHC) Montreal QC Canada; ^4^ L'École de réadaptation de l'Université de Montréal Montréal Canada; ^5^ The Institute of Health Sciences Education McGill University Montréal Canada

**Keywords:** clinical decision‐making, cognitive interviews, content validity, evidence‐based practice, measurement, multidimensional index, psychometrics

## Abstract

**Rationale:**

Clear, interpretable measures that account for linguistic differences are critical to accurately assess rehabilitation clinicians' propensity to integrate research evidence into clinical decision‐making.

**Aims and Objectives:**

To contribute evidence for the clarity and interpretability of a new five‐item bilingual multidimensional index of a rehabilitation clinician's propensity to integrate research evidence into clinical decision‐making.

**Methods:**

This study was conducted in three sequential steps: (1) We conducted a focus group with occupational therapists, physical therapists, and researchers to review the items and response options for clarity, consistency, and interval properties and agree on equivalency in English and French. (2) We conducted cognitive interviews whereby clinicians elaborated on their interpretation of the item, comprehensibility of items, and appropriateness of response options. Accepted modifications were integrated and tested with subsequent participants. (3) We conducted an online survey to validate the English and French equivalency of response options on a 0–100 scale.

**Results:**

During the qualitative revision process (one focus group with seven participants followed by 27 interviews), the index was revised 12 times with substantial modifications to the *use of research evidence* and *attitudes* items.

**Conclusion:**

This study increases the clinical relevance and reduces measurement error of this brief index which can inform on individual or organizational factors influencing a clinician's propensity of integrating research evidence into decision‐making and ultimately improve rehabilitation outcomes.

## 1 Introduction

To optimize quality of care, occupational (OTs) and physical therapists (PTs) are expected to engage in evidence‐based practice (EBP), integrating best available research evidence, clinical expertise, and patient values and preferences when making clinical decisions [1, 2, 3, 4]. Rehabilitation clinicians acknowledge that clinical experience is essential to integrating research evidence into practice [5, 6, 7] and as such, they must make sense of the quality, pertinence and applicability of research evidence using their judgment and tacit knowledge [8, 9].

The tripartite conceptualization of EBP (i.e., research evidence, clinical expertise, and patient values) has traditionally been depicted by the “three circles” model [10, 11, 12, 13]. More recently, refinements and additions to this conceptualization of EBP have been reflected in the inclusion of context (e.g., organizational, economic, professional) to highlight external influences on clinical decision‐making (CDM) [14, 15, 16, 17]. Despite the purported benefits of a CDM approach and the implementation of EBP content into most entry level OT and PT curricula [1, 4] globally, clinicians report difficulties integrating research evidence into practice [18, 19, 20, 21, 22, 23]. Lack of allotted time for activities related to EBP, poor access to research evidence, low confidence in applying research to practice, lack of knowledge that evidence‐based interventions exist, and inadequate equipment to implement new practices are among the reasons for the underutilization of research evidence in practice [7, 18, 19, 20, 23, 24, 25].

Robust measurement practices are needed to identify the factors related to EBP that should be improved or strong areas that must be maintained [26, 27, 28]. Identifying which areas require improvement can inform targeted allocation of resources to support EBP, ultimately improving patient outcomes. A vast selection of questionnaires exist measuring the core factors influencing an OT or PT's likelihood of integrating research evidence [26, 29, 30, 31]. However, there are shortcomings to current EBP measures, such as failure to concurrently measure multiple domains, inappropriate analysis of items derived from ordinal scales, and the unknown relative weight of domains [31].

In previous work, Al Zoubi et al. identified the six most salient domains influencing rehabilitation clinicians' likelihood of integrating research evidence into CDM [28]. One best performing item per domain was chosen to form a brief, multidimensional index in English and French, as described by Roberge‐Dao et al. [32]. The five domains included in the index are: *use of research evidence, self‐efficacy, resources, attitudes*, and *activities related to EBP*. However, as the selected items stem from five different questionnaires, there is inconsistency between items (and response options) in terms of terminology and formulation, which can increase respondent burden and introduce measurement bias. In addition, the English and French versions of these items may present cultural or linguistic discrepancies that introduce systematic differences in scores [33].

The aim of this study was to contribute evidence on the clarity and interpretability of items and response options for a new bilingual measure, the *Propensity to Integrate Research Evidence into Clinical Decision‐Making Index* (PIRE‐CDMI). Specifically, the primary objective was to review and revise the included items in the prototype index in English and French. The secondary objective was to estimate the equivalency of response option labels in both languages.

## 2 Methods

**Figure 1:**
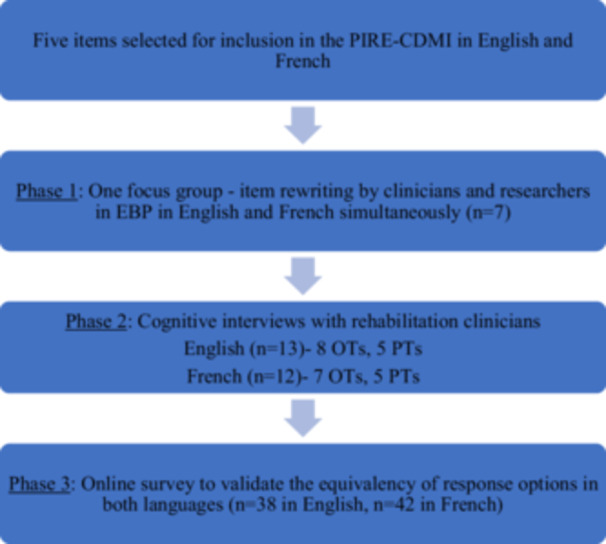
Overview of the item revision process for the Propensity to Integrate Research Evidence into Clinical Decision‐Making Index (PIRE‐CDMI).

This study involved a three‐phase qualitative review process illustrated in Figure 1. Ethics approval was obtained from The Faculty of Medicine and Health Sciences Institutional Review Board at McGill University for all phases of this study before commencement and recruitment.

### 2.1 Phase 1: Focus Group

#### 2.1.1 Focus Group Participants

Practicing OTs and PTs and EBP researchers (defined as researchers having experience in EBP research and having published a minimum of one EBP‐related publication) were recruited purposefully from the research team's networks to participate in a 90‐min online focus group. The pool of participants was expected to be bilingual and have equal representation of both French and English native speakers.

#### 2.1.2 Focus Group Process

The focus group aimed to review and revise the items and response options of the prototype PIRE‐CDMI for clarity, consistency, and interval properties and arrive at a consensus on modifications needed to have equivalent versions in English and French [10, 34]. Participants were asked to establish equivalence in both languages such that the items, instructions, and response options were conceptually (i.e., do people in both groups see the concept in the same way) and semantically (i.e., the meaning attached to words in an item) comparable [34, 35]. Consenting participants were sent the items with a reminder of the study aim one week before the focus group.

The online focus group was conducted via Zoom and structured as follows: (1) welcome and overview of the study; (2) objectives, instructions, and an example for item rewriting; (3) breakout rooms with two individuals per room for five min to attempt to review one item; (4) attend to any questions that arose during breakout rooms; (5) collective item rewriting exercise using a shared screen.

**Figure 2:**
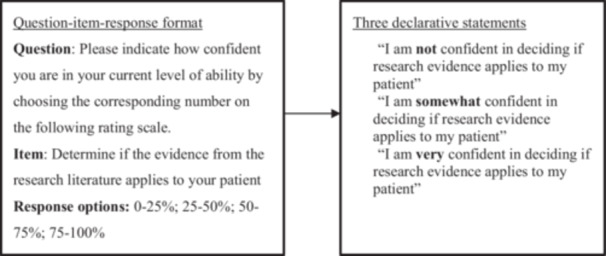
Example of the item rewriting exercise in the focus group.

During the item rewriting exercise, the moderator (first author JRD) structured the discussion, and a note‐taker recorded the suggested modifications on a shared online document. Participants were asked to rewrite items from question‐item format into declarative statements from the perspective of a clinician (see Figure 2 for an example of this process). Probing questions included: (1) How would you rewrite this item into a declarative statement? (2) Is the wording clear, and if not, how would you change it? (3) How difficult would it be for OTs and PTs to answer these items? For each item, the French translation was discussed simultaneously. Once every item was discussed, the moderator asked participants to verify that the overall index was coherent in terms of wording and length, that items read well together, and that everyone agreed on the final set of items. After the focus group, the research team (consisting of bilingual EBP researchers in rehabilitation) reviewed the suggested final set of items and resolved any outstanding discrepancies.

### 2.2 Phase 2: Cognitive Interviews

#### 2.2.1 Cognitive Interview Participants

New participants were recruited for cognitive interviews. Clinicians were eligible to participate in a cognitive interview if they were (1) practicing OTs and PTs in Canada; (2) native French‐ or English‐speaking; and (3) practicing for a minimum of one year. The research team used social media (Twitter and Facebook) and the University's newsletter to advertise the project. Interested participants entered their contact information in an online form. A member of the research team contacted them to provide more information on the study. Participants gave informed consent to participate in the research.

#### 2.2.2 Cognitive interview process

Cognitive interviews were conducted by the first author (JRD) by telephone or Zoom with potential respondents of the PIRE‐CDMI (i.e., OTs and PTs) to identify and rewrite any problematic items to increase the overall readability, functioning, and interpretability of the measure [34, 36, 37, 38, 39, 40]. All interviews were audio recorded and lasted 15−30 min.

Participants received the updated PIRE‐CDMI at least one day before the scheduled interview. As presented in Table 1, the interviewer used the verbal probing method to elicit participants' comprehension of all five items by asking specific questions regarding meaning, clarity, and interpretation of items [38, 41]. These questions were adapted from a study using similar methods to develop a preference‐based index for multiple sclerosis [42]. Participants were encouraged to think aloud while going through the measure, providing insight into how a participant perceived and interpreted the items [41].

**Table 1:** Cognitive interview probing questions.

**Items** What does this statement mean to you? In your own words, what do you think this statement is saying? Were these statements easy to understand? Are there any words in this statement that are not clear or do not work well? How would you change the wording to make it clearer?
**Response options**
What do you think about the three options? How would you make the three options clearer?
**Overall impression of the measure**
Do you have any comments on the measure as a whole? Is there anything that you would change in the measure? Would you change anything with the visual presentation?

#### 2.2.3 Analysis of Cognitive Interviews

English and French interviews were conducted in parallel to avoid prioritizing a language. After each day of interviews, the interviewer reviewed comments and revised problematic items based on participants' suggestions. The research team reviewed the feedback before implementing the change and proposed suggestions based on best practices of item development, such as simple items that express a single idea, using common vocabulary, and avoiding colloquialisms [34, 39, 43]. Changes were implemented in both languages simultaneously, when applicable. The revised PIRE‐CDMI was then tested on the next round of participants. Interviews were conducted until no changes suggested by three consecutive participants were necessary [42, 44].

### 2.3 Phase 3: Survey

Given Canada's linguistic diversity, methods to ensure the equivalence of questionnaire versions in the two official languages were warranted to decrease systematic differences between language groups. Validation of translations through quantitative response scaling can contribute to demonstrating that respondents are interpreting items in a similar fashion and consist of asking respondents to denote the position of response options on a visual analog scale (VAS) (i.e., a line from 0 to 100) and to compare ratings between languages. These methods have been used in previous studies [45, 46].

#### 2.3.1 Survey Respondents

This phase consisted of a cross‐sectional online survey to generate additional evidence on the equivalency of PIRE‐CDMI response option labels in English and French. The target population was healthcare professionals and students that (1) were native English or French speakers and (2) worked or studied in a healthcare professional or graduate program in Canada. We used convenience sampling and did not exclude respondents based on profession or level of training because the nature of the survey was such that respondents needed only to have the abilities to interpret common words and rate response option labels on a numerical scale. We recruited through social media, and interested respondents were invited to follow a link with study information, a consent statement, and an invitation to start the survey in their language of choice. No identifying or sociodemographic information data were collected.

#### 2.3.2 Survey Procedure and Analysis

The survey was piloted with seven graduate students who were also practicing clinicians in rehabilitation. Modifications were integrated to improve survey clarity and task comprehension. The survey was open from November to December 2021.

Each PIRE‐CDMI item was associated with three response option labels. Respondents were asked to indicate the position of each of the three response option labels on a 0−100 VAS between two anchors. Response option labels belonging to the same set appeared sequentially on a single page. This method has been previously reported for health‐related quality of life measures such as the SF‐36 [45] and EuroQol‐5d [46]. Specifically, participants were asked: “On the line, where would you position each of the three response option labels between [the bottom anchor] and [the top anchor]?” Appendix I presents the three response option labels for each item and associated response anchors. The full PIRE‐CDMI item was also stated on the same page as the response set to provide context.

It was hypothesized that respondents would position the lowest response option label closest to the zero anchor, the middle response option label in the middle, and the highest response option label closest to the 100 anchor. Post hoc analyses were conducted to remove respondents who likely misunderstood the task reflected in having either: (1) more than two sets of disordinal response patterns; (2) the same rating for all three response options for one item or more; or (3) two or more extreme outlier ratings (0 or 100). Multiple linear regression was used to estimate the extent to which VAS ratings per item (0−100 scale) depended on language (English, French), response option (low, middle, high), and the interaction between language and response option. The normal probability plot of standardized residuals was visually examined.

## 3 Results

### 3.1 Focus Group Item Rewriting

Four PTs and three OTs, all doctoral candidates with research experience in EBP, participated in the 90‐min focus group. Each five question‐item‐response set was transformed into five sets of three declarative statements that were then clarified and harmonized in English and French (version 2 of the PIRE‐CDMI). Participants agreed that response options including the word “never” (e.g., “I never integrate research evidence”) were undesirable because choosing these options would make them be perceived as incompetent. Given that clinicians would not opt for these options, the participants removed the word “never” from the *use of research evidence* and *activities* items. For the *activities* item, focus group participants communicated that it was important not to confine research evidence to scientific articles; they suggested replacing “read research” with “consult research evidence”. Further, participants agreed that omitting the verb “reading” was more inclusive of individuals with visual impairments. In French, multiple terms were proposed for “research evidence” (e.g., évidences ou preuves scientifiques, données issues de la recherche), but the agreed upon term was *données probantes*, which was said to be most employed and recognized among clinicians.

Three issues remained unresolved after the focus group and were subsequently discussed within the research team. Modifications were made to the items before starting cognitive interviews (version 3). First, it was unclear whether “patient” or “client” was preferable, as the terms are often used interchangeably in rehabilitation contexts depending on the setting and the population. The research team agreed to use “patient” consistently and added a footnote to explain the interchangeable nature (N.B. the final wording of items did not include the words “patient” or “client”). Second, participants did not agree on whether to use “organization” or “clinical setting”. The research team modified the item to focus on the broad availability of resources and refrained from using either term. Finally, participants could not come to a consensus between “willing” and “inclined” in the *attitudes* item, as they were compelled to rate *attitudes* items highly; although clinicians may be willing or inclined to use evidence, they may not actually do so in practice. Participants reported that “willing” and “inclined” did not have commonly‐used equivalent French translations that would be suitable for a self‐report measure (*enclin à* or *disposé à* are not commonly used words). Thus, the *attitudes* item was reframed from “I am willing to use EBP” to “it is worth the effort to [use EBP]”. Appendix II reports the step‐by‐step changes at each step of the qualitative rewriting process.

### 3.2 Item Modifications From the Cognitive Interview Process

Twenty‐four individual cognitive interviews were conducted with 10 PTs and 14 OTs in Canada (13 native English speakers, 12 native French speakers; one bilingual participant provided feedback in both languages). Appendix III presents an overview of the item evolution process during cognitive interviews.

The *self‐efficacy* item underwent three iterations. From the initial item, “I am (very confident/somewhat/not very) confident in my ability to integrate evidence into my intervention plan”, the word “integrate” was replaced with “apply” to be more action oriented. “Intervention plan” was first replaced with “clinical cases” to include clinicians who solely perform assessments. “Clinical cases” was then simplified to “practice” to avoid any confusion associated with the variability in clinical cases. Finally, the response option label “very confident” was changed to “confident” because participants stated it was difficult to endorse being very confident with one's ability to apply research evidence to practice. In the final version, two response option labels (“somewhat” and “not very” confident) were not translated exactly to French (*moyennement* and *peu confiant)*.

The item on *use of research evidence* underwent three iterations. At the start of the cognitive interviews, this item consisted of asking respondents about the source of information, either research evidence, colleagues, or clinical experience, that they would seek when faced with a practice uncertainty. Participants found this item particularly difficult to answer because it was dependent on the case at hand (e.g., the availability of evidence for a clinical diagnosis or patient values) and the organizational context (e.g., whether colleagues were available and/or had experience related to the case). Clinicians reported that they often used a combination of all three sources and that it was difficult to select one to describe their typical behavior. The final wording focused on the frequency of using research evidence when faced with a practice uncertainty, which avoids the conflicting response options of colleagues and clinical experience. Finally, an asterisk was added to define practice uncertainty as “a situation in which there is a gap in your knowledge relating to a clinical decision”.

With six versions, the *attitudes* item underwent the most iterations. At the start of the cognitive interviews, respondents were asked the extent to which incorporating evidence into practice was worth the effort. Participants suggested that the item not contain the connotation of “worth the effort” because it was (1) prone to social desirability bias (e.g., participants felt pressured to respond with the highest level) and (2) did not translate well into French (e.g., *cela vaut l'effort* or *cela vaut la peine*). The item was modified to focus on EBP requiring effort (e.g., “It requires [little/some/a lot of] effort to integrate research evidence into practice”). The response option label “some (effort)” was changed to “moderate (effort)” to clarify the middle level response, and the words “(requires… effort) for me” were added to clarify the intent of eliciting the individual's perception of effort rather than a general belief. In French, the direct translation of “it requires little effort for me to…” is *cela me requiert peu d'efforts pour*… which was problematic for two reasons. First, starting a sentence in French with *cela* was too informal. Second, the verb *requiert* was too formal. The structure of the French sentence was changed to place the object (*intégrer les données probantes dans ma pratique*) before the verb and replace *me requiert* with *me demande*.

The *resources* item underwent three iterations. The initial item was “I feel that I have [the/only some of/do not have] the necessary resources to integrate research evidence into my practice”. Participants suggested omitting “I feel that …” and questioned which resources the item referred to. An asterisk was added to clarify meaning and enumerate examples of resources facilitating EBP. In French, participants preferred the verb *je possède (les ressources nécessaires)* to *j'ai (les ressources nécessaires)*. For one response option label (“some of”), the French wording was not an exact translation (*une partie des*).

Finally, the *activities* item underwent the least number of modifications. The only modification consisted of changing the words “consult research evidence” to “keep up to date with research evidence”. Participants interpreted the initial item as the frequency of using evidence in their practice, which was already reflected in the *use of research evidence* item. The revised item reflects the concept of staying up‐to‐date with research evidence as an activity outside of routine CDM. Though three of the 24 participants suggested explicitly describing and quantifying the three adverbs (regularly, occasionally, and rarely), the research team decided to avoid this, as there is no agreed upon best practice for behavioral frequency of consulting the literature. By providing these three response options without specifying the exact range, the research team intended to capture clinicians' self‐reports relative to their temporal understanding of keeping up‐to‐date with research evidence in their field. In French, “keeping up to date with research evidence” did not translate exactly, so the following modification was retained for conceptual equivalence: *se tenir à jour quant aux données probantes*.

The initial instructional prompt was “For each group of statements, select ONE statement which best applies to you. Please respond as honestly as possible”. The prompt was modified three times to give the final version, “Please select ONE statement from each box which best reflects your current practice and context.”

The visual presentation of the measure was improved following participant suggestions. Specifically, the lettering of each response option was bolded to facilitate discriminating between levels. It was also suggested to number the five items (1−5) and letter the three response options (a, b, c) to reduce cognitive burden involved in completing the index. The final version of the PIRE‐CDMI in English and French are provided in Appendix SIV.

### 3.3 Scaling of Response Option Labels

**Figure 3:**
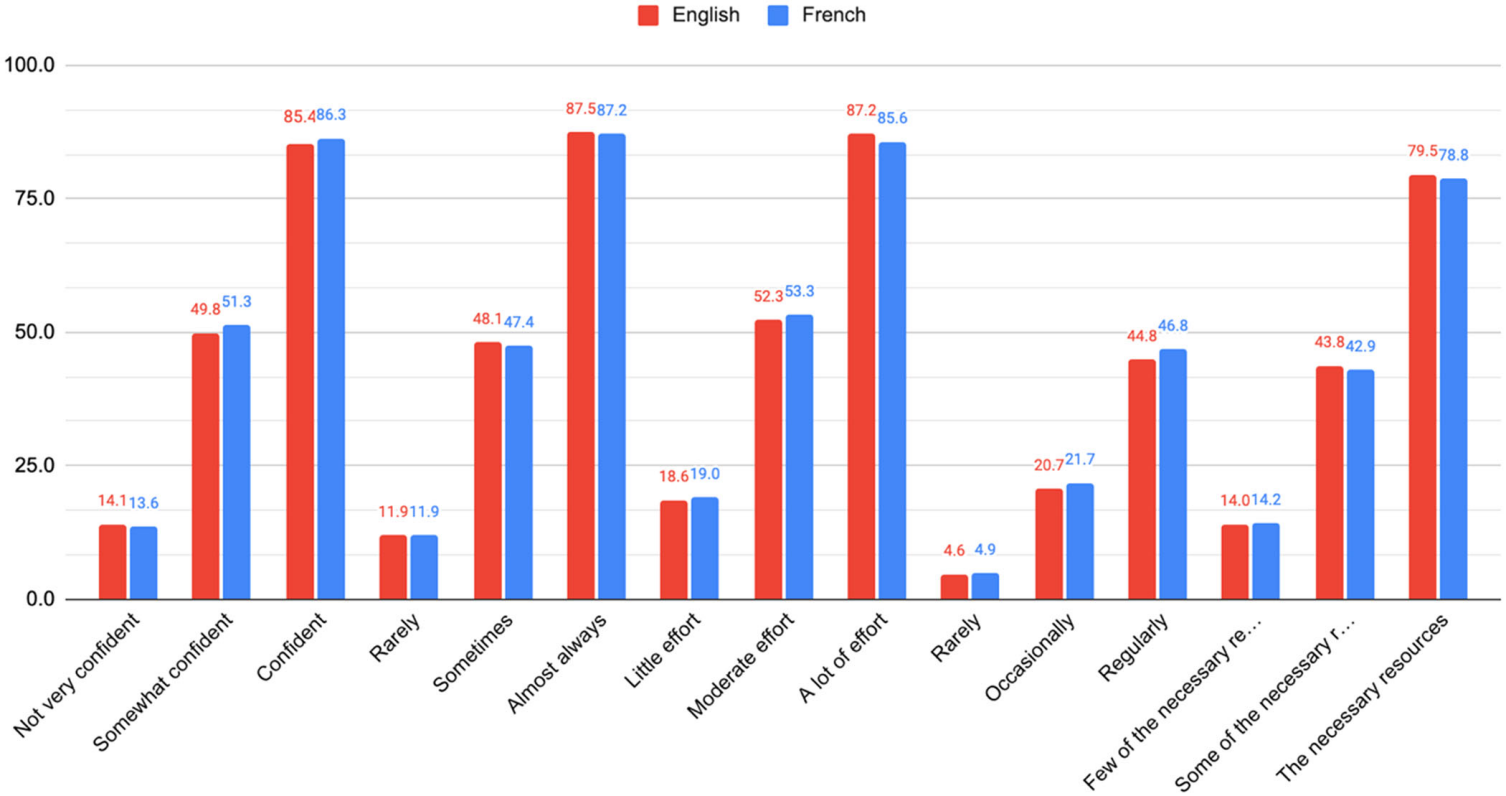
Histogram illustrating the frequency distribution of mean ratings of response option labels on a 0−100 scale for the five PIRE‐CDMI items in English and French.

Among the 129 individuals who started the online survey, 60 were Canadian French speakers (46%) and 69 were English speakers (54%). Of these, 42 Francophones (32%) and 38 Anglophones (30%) were included for analysis. The rest were excluded due to incomplete surveys (*n* = 25, 19%) and task miscomprehension (*n* = 24, 19%). Descriptive results for the rating of the five response option sets by the 80 respondents are presented in Table 2. The ordinal nature of the ratings of response sets is illustrated in Figure 3. Multiple linear regression results did not suggest any important main effects of language on scores for the five items, nor any important interaction of language and response option. Aside from a few outliers in all items, the residuals were normally distributed.

**Table 2:** Descriptive statistics for the rating of response options on a 0–100 scale (*n* = 80).

	Mean (SD)	Min	Max	Mean (SD) in English (*n* = 38)	Mean (SD) in French (*n* = 42)
**Item 1**: I am _____ in my ability to apply research evidence to practice. **Anchors**: No confidence/Full confidence					
Not very confident	13.8 (5.2)	0	28	14.1 (3.7)	13.6 (6.2)
Somewhat confident	50.6 (6.6)	30	74	49.8 (6.3)	51.3 (7)
Confident	85.9 (5.9)	70	100	85.4 (4.7)	86.3 (6.9)
**Item 2**: When faced with a practice uncertainty, I ____ use research evidence. **Anchors**: None of the time/All of the time					
Rarely	11.9 (4.9)	0	26	11.9 (3.5)	11.9 (5.9)
Sometimes	47.7 (7.9)	25	75	48.1 (7.1)	47.4 (8.7)
Almost always	87.3 (5.3)	70	100	87.5 (4.1)	87.2 (6.3)
**Item 3**: It requires _____for me to integrate research evidence into practice. **Anchors**: No effort/Full effort					
Little effort	18.8 (5)	0	27	18.6 (4.5)	19 (5.4)
Moderate effort	52.9 (5.7)	38	70	52.3 (3.5)	53.3 (7.1)
A lot of effort	86.4 (4.8)	75	100	87.2 (4.1)	85.6 (5.2)
**Item 4:** I _____ keep up to date with research evidence. **Anchors**: 0 days/month/30 days/month					
Rarely	4.8 (4.9)	0	17	4.6 (4.4)	4.9 (5.3)
Occasionally	21.3 (11.3)	3	60	20.7 (7.8)	21.7 (13.7)
Regularly	45.9 (18.2)	13	100	44.8 (16.2)	46.8 (20)
**Item 5:** I have _____ to integrate research evidence into my practice. **Anchors**: None/All imaginable resources					
Few of the necessary resources	14.1 (4.6)	3	25	14.0 (3.8)	14.2 (5.2)
Some of the necessary resources	43.4 (6)	20.0	56.0	43.8 (6.5)	44 (9.3)
The necessary resources	79.2 (7.2)	60.0	100.0	79.5 (7.2)	79.3 (8)

Abbreviation: SD, standard deviation.

## 4 Discussion

This study describes a robust item revision and rewriting process of an index measuring the propensity of rehabilitation clinicians to integrate research evidence into CDM. This three‐step approach to qualitative revision has not been previously reported in the EBP literature. Overall, the index (PIRE‐CDMI) underwent 12 iterations to increase the clinical relevance and reduce measurement error, with important changes made to the *use of research evidence* and *attitudes* items.

The *use of research evidence* item changed considerably. Early iterations relating to (1) the frequency of integrating the three EBP pillars (research evidence, clinical expertise, and patient preferences) and (2) clinicians' primary source of knowledge (research evidence, colleagues, and clinical experience) failed to produce useful information because clinicians attested to integrating all three pillars of EBP into CDM and relying on all proposed sources of knowledge to various extents. In fact, the tripartite definition is foundational to how rehabilitation clinicians conceptualize EBP [47]. However, asking clinicians to select their most relied upon pillar of EBP or to determine the frequency at which they integrate the three components is anathema to the reality of CDM, as these elements are inextricably intertwined [21]. Further, asking clinicians to select the most relied‐upon source of knowledge in a measure relating to EBP appears to introduce high levels of social desirability bias [48, 49]. For example, a respondent may interpret the desirable source of information to be “research evidence” and “colleague” or “clinical experience” to be undesirable sources. The item formulation may have inadvertently implied that consulting a colleague or relying on clinical experience is ill‐advised when, in fact, these sources of knowledge are foundational to being a competent, reflexive, and evidence‐based clinician [9, 22, 47, 50]. Though plurality of knowledge in CDM is increasingly invoked in the field of EBP [51], rehabilitation clinicians continue to identify challenges related to the integration of research evidence into CDM, signaling a deep‐rooted need for support with this component of EBP [19, 22, 24, 25].

The notion of clinical uncertainty was introduced into the *use of research evidence* item to contextualize the behavior of seeking and using research evidence in CDM. The addition of contextual cues in items has been said to increase the validity of responses, notably when the behavior has an element of automaticity [52]. CDM often relies on automatic and intuitive reasoning rather than analytical reasoning, a phenomenon which is hypothesized to become stronger over time [53, 54]. This clinical uncertainty allows clinicians to tap into their analytical reasoning and can be compared to *the event* proposed in the reflective practice literature, defined as “an event that occurs in everyday practice […] that leaves the occupational therapist with the urge to revisit it to make sense of it for the benefit of his or her future practice” [55]. The need to include clinical uncertainty in this item is further reinforced by a possible mechanism whereby, over time, research‐based knowledge becomes consolidated into tacit or experiential knowledge. In such cases, it may no longer be distinguished as *research evidence* but rather transformed into expert practice adapted to the practice context [56, 57]. Correspondingly, it may be difficult for clinicians to discern how frequently they use research evidence on a day‐to‐day basis. Lastly, consulting research evidence in everyday practice may not be realistic or desirable, as it could conceal other professional difficulties such as low confidence in one's clinical reasoning abilities [58]. Thus, the more compelling question is not so much whether clinicians consult formal sources of evidence every day but whether they do so when confronted with a gap in their knowledge.

Many modifications were made to the *attitudes* item to remedy the social desirability bias reported by participants. Despite changes to the item, participants, most of whom were educated in Canada, continued to report that EBP was a desirable process and were compelled to select the highest response option for attitudes. The evidence demonstrating the relationship between attitudes towards EBP and EBP behavior is inconclusive. While some studies have suggested that holding positive attitudes towards EBP is an important precursor to EBP behavior [59, 60, 61, 62, 63], others have demonstrated that they do not translate into effective EBP behavior [18, 19, 20, 64, 65, 66, 67]. We postulate that measuring attitudes towards EBP may not be useful in the context of this brief index given that (1) the relationship between attitudes and EBP behaviors is uncertain; (2) it is well‐established that rehabilitation clinicians are generally convinced of the value of EBP and believe it to be a desirable and necessary process [18, 19, 20, 65, 67, 68, 69]; and (3) value‐laden items which can prejudice respondents should be omitted from measures [34] and attitudes are inherently value‐laden. Furthermore, when assessing attitudes for predicting behaviors, it is recommended to avoid measuring attitudes towards a general concept and to focus on specific behaviors [70]. Using *effort* instead of *attitudes* circumvents asking clinicians whether they consider EBP to be valuable and highlights the perceived cost of integrating research evidence into practice [71]. *Effort* is defined by the Cambridge Dictionary as “physical or mental activity needed to achieve something”. Social psychology and behavioral theorists have identified effort as largely contributing to behavioral motivation [72, 73, 74]. People are less likely to engage in a behavior if it requires a large amount of effort [75]; several studies have shown that rehabilitation clinicians perceive the enactment of EBP as being effortful [21, 22, 65].

One noteworthy modification to the *activities* item was the inclusion of various sources of research evidence beyond scientific articles. As such, one could associate keeping up‐to‐date with research evidence with leading or assisting in a journal club, reading email subscription alerts, or gaining research‐based knowledge from a colleague. This departure from formal sources of research‐based knowledge is more aligned with the behaviors and preferences of rehabilitation clinicians, who favor informal, quick methods of gaining research evidence and tend to keep up with research through a variety of informal sources [7, 21, 22, 45, 76, 77, 78]. The process by which rehabilitation clinicians rely on colleagues for research‐based knowledge is starting to gain recognition in the EBP literature as a beneficial mechanism of EBP [22, 76, 79, 80]. This is a promising avenue for future research.

In the third and last step of this study, the effect of language on VAS ratings was trivial, supporting the equivalency of the response option labels in English and French for all items. The distribution of ratings also demonstrated the ordinal consistency of response options and the quasi‐interval nature of the scales (i.e., equally spaced response options). The *keeping up to date* item had the largest variation in ratings between languages and within the same language; however these differences were less than five on 100 and are considered negligeable [81]. A possible explanation for this variability may be the subjective nature of the descriptions of behavioral frequency (potentially dependent on the area of practice) and lack of consensus on how often rehabilitation clinicians should consult the literature. As knowledge is produced at different rates for different areas of practice, and the fact that this index is meant to be used in various settings, no explicit frequency denominator was attributed to this item. For instance, “regularly” could mean once every two months for a clinician in stroke rehabilitation or once per year for a clinician in palliative care. Our intent with this item was to capture the respondent's self‐rating relative to their understanding of what “regularly”, “occasionally”, and “rarely” mean in their field of practice and relative to their perception of what is feasible given their clinical reality.

### 4.1 Strengths and Limitations

A strength of this study is the rigorous multi‐phased qualitative review process which included target end‐users and EBP researchers. In developing the response option scaling survey, we aimed to provide adequate guidance to maximize respondents' comprehension of the task. Pilot testing enabled us to add examples and clarify the instructions. Still, given that 25 individuals did not complete the survey (19%) and that an additional 24 had to be excluded due to apparent miscomprehension of the task (19%), this exercise may have been perceived as difficult and burdensome, a finding also reported by others in the context of a valuation exercise for the EQ‐5D [82]. The task involved an unfamiliar method of placing response option labels on a 0−100 scale, which required abstract reasoning. Given the lack of available demographic data, it is impossible to discern who misunderstood the task. Comprehension may have been improved with a short instructional video.

Before deploying the PIRE‐CDMI, there remains the important developmental step of estimating the relative weights of each dimension‐level. This study, which the research team has started, will allow for the generation of a more accurate total score that takes into consideration end‐users' perceived relative contribution of dimensions on the overall construct of propensity to integrate research evidence into CDM. We acknowledge that the initial mathematical properties of the prototype PIRE‐CDMI established in previous research [32] may have changed due to item rewriting. While this must be confirmed in future testing, our findings pertaining to the quasi‐interval spacing of response option labels gives us reason to believe that the interval properties of the scale are still valid. Due to important linguistic differences between different countries, we suggest undergoing a thorough cultural adaptation and reassessment before using the PIRE‐CDMI with English and French‐speaking individuals outside of Canada.

Finally, while some authors have stated that short scales are a limitation and can compromise the validity and reliability of inferences drawn from a measure [83, 84], others have found value in the efficiency of short scales [85, 86]. There exists a delicate trade‐off between scale comprehensiveness and feasibility. Given the resource‐strained healthcare context, the aim was to create a short index capable of rapidly estimating elements of EBP requiring improvement. This measure can be used as an efficient global outcome measure of a clinician's propensity to integrate research evidence into CDM for research purposes and professional self‐reflection, which may then be complemented with more comprehensive and lengthier measures. Intervention strategies can then be developed to target the specific areas requiring support.

## 5 Conclusion

The three consecutive phases described in this paper illustrate a rigorous approach to developing a brief multidimensional index of propensity to integrate research evidence into CDM in rehabilitation that is coherent, clear, and relevant to Canadian OTs and PTs. A focus group and cognitive interviews led to important item modifications in English and French to minimize ambiguity, measurement bias, and cognitive burden on respondents. Finally, response option labels in English and French were found to be equivalent through a cross‐sectional online survey wherein response option labels were compared on 0−100 scales in both languages. It is hoped that the use of this practical index will help identify research and practice needs, better support clinicians, and improve the quality of rehabilitation care.

## Conflicts of Interest

The authors declare no conflicts of interest.

## Supporting information

Roberge‐Dao Indexclarity Appendices.

## Data Availability

Data available on request due to privacy/ethical restrictions.
