# Supplementary material for: Improving Clarity and Interpretability of Items in a Bilingual Index of Propensity to Integrate Research Evidence Into Clinical Decision‐Making in Rehabilitation
Source: J Eval Clin Pract. 2025 Jul 9;31(5):e70196. doi: 10.1111/jep.70196 (PMC12239708; doi:10.1111/jep.70196)
Supplement: Supplementary file 1 — Roberge‐Dao Indexclarity Appendices. [file JEP-31-0-s001.docx]

## Appendices

### Appendix I. Five PIRE-CDMI response sets

| **Dimension** | **Response options** | **Response anchors** |
| --- | --- | --- |
| Use of research evidence | Almost always  Sometimes  Rarely | None of the time / All of the time |
| Self-efficacy | Confident  Somewhat confident  Not very confident | No confidence / full confidence |
| Resources | Have the necessary resources  Have some of the necessary resources  Have few of the necessary resources | None / All imaginable resources |
| Attitudes - Effort | Little effort  Moderate effort  A lot of effort | No effort / Full effort |
| Activities related to EBP - Keeping up to date | Regularly  Occasionally  Rarely | 0 days/month /  30 days/month |

### Appendix II. Step-by-step changes at each step of the item rewriting process in both languages for each item

**English version of the index**

| Item: Self-reported use of research evidence | | | |  |
| --- | --- | --- | --- | --- |
| Version 1 | How often have you done each of the following activities in the past month? | Decide on an appropriate course of action based on integrating the research evidence, clinical judgment and patient or client preferences? | 0 (Never) / 1 (One time or more) |  |
| Focus group | Changed the initial item structure to three declarative statements. Changed "[integrating the 3 pillars]" to "integrate research evidence". Changed "decide on an appropriate course of action" to "into the care plan". | | | |
| Version 2 | I regularly integrate research evidence into the care plan. | I occasionally integrate research evidence into the care plan. | I rarely integrate research evidence into the care plan. |  |
| Research team | Changed item to elicit the first source of information when faced with a practice uncertainty. | | | |
| Version 3 | When faced with a practice uncertainty, I rely on research evidence. | When faced with a practice uncertainty, I rely on my colleagues. | When faced with a practice uncertainty, I rely on my clinical experience. |  |
| Cognitive interviews | Changed the sentence structure. | | | |
| Version 4 | I first rely on **research evidence** when faced with a practice uncertainty. | I first rely on **my colleagues** when faced with a practice uncertainty. | I first rely on **my clinical experience** when faced with a practice uncertainty. |  |
| Version 5 | Idem |  |  |  |
| Cognitive interviews | Added an asterisk to define what we mean by "practice uncertainty" | | | |
| Version 6 | I first rely on **research evidence** when faced with a practice uncertainty. | I first rely on **my colleagues** when faced with a practice uncertainty. | I first rely on **my clinical experience** when faced with a practice uncertainty. | **Uncertainty: a situation in which there is a gap in your knowledge relating to a clinical decision* |
| Version 7 | Idem |  |  |  |
| Version 8 | Idem |  |  |  |
| Version 9 | Idem |  |  |  |
| Version 10 | Idem |  |  |  |
| Cognitive interviews | To decrease variability due to clinical population and context, the item was modified to focus on frequency of using research evidence when faced with a practice uncertainty. | | | |
| Version 11 | When faced with a practice uncertainty*, a) I **almost always** use research evidence. | b) I **sometimes** use research evidence. | c) I **rarely** use research evidence. | **Uncertainty: a situation in which there is a gap in your knowledge relating to a clinical decision* |
| Version 12 | Idem |  |  |  |
| Item: Self-efficacy | | | |  |
| Version 1 | Please indicate how confident you are in your current level of ability by choosing the corresponding number on the following rating scale. | Determine if the evidence from the research literature applies to your patient | 0 (0−25%) / 1 (25−50%) / 2 (50−75%) /3 (75−100%) |  |
| Focus group | Changed the initial item structure to three declarative statements. Omitted “in your current level of ability” to shorten the sentence. Changed “evidence from the research literature” to “research evidence” to be inclusive to other sources of research evidence. | | | |
| Version 2 | I am confident in deciding if research evidence applies to my patient*. | I am somewhat confident in deciding if research evidence applies to my patient*. | I am not confident in deciding if research evidence applies to my patient*. |  |
| Research team | Changed "deciding if research evidence applies to my patient" to "in my ability to integrate research evidence into my intervention plan". Added "very" to first and last level. | | | |
| Version 3 | I am very confident in my ability to integrate research evidence into my intervention plan. | I am somewhat confident in my ability to integrate research evidence into my intervention plan. | I am not very confident in my ability to integrate research evidence into my intervention plan. |  |
| Version 4 | I am **very confident** in my ability to integrate research evidence into my intervention plan. | I am **somewhat confident** in my ability to integrate research evidence into my intervention plan. | I am **not very confident** in my ability to integrate research evidence into my intervention plan. |  |
| Cognitive interviews | Changed "integrate" to "apply". Changed “intervention plan” to “clinical cases”. | | | |
| Version 5 | I am **very confident** in my ability to apply research evidence to clinical cases. | I am **somewhat confident** in my ability to apply research evidence to clinical cases. | I am **not very confident** in my ability to apply research evidence to clinical cases. |  |
| Version 6 | Idem |  |  |  |
| Version 7 | Idem |  |  |  |
| Version 8 | Idem |  |  |  |
| Cognitive interviews | Changed “very confident" to "confident". | | | |
| Version 9 | I am **confident** in my ability to apply research evidence to clinical cases. | I am **somewhat confident** in my ability to apply research evidence to clinical cases. | I am **not very confident** in my ability to apply research evidence to clinical cases. |  |
| Cognitive interviews | Changed “clinical cases” to “practice”. | | | |
| Version 10 | I am **confident** in my ability to apply research evidence to practice. | I am **somewhat confident** in my ability to apply research evidence to practice. | I am **not very confident** in my ability to apply research evidence to practice. |  |
| Version 11 | Idem |  |  |  |
| Version 12 | Idem |  |  |  |
| Item: Resources | | | |  |
| Version 1 | Please indicate your level of agreement with the following statements: | My organization supports best practice | 0 (strongly disagree /disagree) / 1 (neutral) /2 (agree) /3 (strongly agree) |  |
| Focus group | Changed the initial item structure to three declarative statements. “Best practice” was changed to “evidence-based practice”. | | | |
| Version 2 | My [organization/clinical setting] supports evidence-based practice. | My [organization/clinical setting] somewhat supports evidence-based practice. | My [organization/clinical setting] does not support evidence-based practice. |  |
| Research team | Changed from "my organization supports best practice" to "I feel that I have the necessary resources to …" | | | |
| Version 3 | I feel that I have the necessary resources to integrate research evidence into my practice. | I feel that I only have some of the necessary resources to integrate research evidence into my practice. | I feel that I do not have the necessary resources to integrate research evidence into my practice. |  |
| Cognitive interviews | Omitted "I feel that". Omitted "I only have some". Changed "I do not have" to "I have few". Added the examples of resources in parentheses. | | | |
| Version 4 | I **have the necessary resources** (e.g., [paid time to consult the evidence, access to journals, therapeutic material...] to integrate research evidence into my practice. | I **have some of the necessary resources** to integrate research evidence into my practice. | I **have few of the necessary resources** to integrate research evidence into my practice. |  |
| Cognitive interviews | Added an asterisk with examples of resources. | | | |
| Version 5 | I have **the necessary resources*** to integrate research evidence into my practice. | I have **some of the necessary resources*** to integrate research evidence into my practice. | I have **few of the necessary resources*** to integrate research evidence into my practice. | *Examples: paid time to consult the evidence, access to journals, therapeutic material... |
| Version 6 | Idem |  |  |  |
| Cognitive interviews | Added “computer” to the examples. | | | |
| Version 7 | Idem |  |  | *Examples: paid time to consult the evidence, access to journals, access to computer, therapeutic material... |
| Version 8 | Idem |  |  |  |
| Version 9 | Idem |  |  |  |
| Version 10 | Idem |  |  |  |
| Version 11 | Idem |  |  |  |
| Version 12 | Idem |  |  |  |
| Item: Attitudes - Effort | | | |  |
| Version 1 | Please indicate your level of agreement with the following statements: | I am willing to use new and different types of clinical interventions (e.g., assessment, treatment) developed by researchers to help my patients/clients. | 0 (strongly disagree)/1 (disagree)/ 2 (neutral)+ 3 (agree) / 4 (strongly agree) |  |
| Focus group | Changed the initial item structure to three declarative statements. Replaced the ending “to help my patients/clients” with “for my patient’s care plan”. Replaced “to use new and different types of clinical interventions (e.g., assessment, treatment) developed by researchers” with “to use EBP” to simplify the item. | | | |
| Version 2 | I am [willing/inclined] to use EBP for my patient’s* care plan. | I am [somewhat willing/somewhat inclined] to use EBP for my patient’s* care plan. | I am [not willing/not inclined] to use EBP for my patient’s* care plan. |  |
| Research team | Changed from "I am willing to use EBP…" to the notion of "worth the effort …" to diminish social desirability bias. | | | |
| Version 3 | Incorporating evidence into my practice is definitely worth the effort. | Incorporating evidence into my practice is somewhat worth the effort. | Incorporating evidence into my practice is not worth the effort. |  |
| Cognitive interviews | Switched the structure of the sentence. Added "(not) really (worth the effort)". Added parentheses with specification after practice. | | | |
| Version 4 | It is **definitely worth the effort** to incorporate research evidence into my practice (i.e., assessment and/or intervention plan). | It is **somewhat worth the effort** to incorporate research evidence into my practice. | It is **not really worth the effort** to incorporate research evidence into my practice. |  |
| Cognitive interviews | Changed "incorporate" to "integrate" for consistency throughout the measure. Deleted the example “because practice is used before without specification". Changed “worth THE effort” to “worth MY effort”. | | | |
| Version 5 | It is **definitely worth my effort** to integrate research evidence into my practice. | It is **somewhat worth my effort** to incorporate research evidence into my practice. | It is **not really worth my effort** to incorporate research evidence into my practice. |  |
| Version 6 | Idem |  |  |  |
| Cognitive interviews | Removed the superlative "definitely" from first response option. Changed "my (effort)" to "the (effort)". | | | |
| Version 7 | It is **worth**the effort to integrate research evidence into my practice. | It is **somewhat**worth the effort to incorporate research evidence into my practice. | It is **not really** worth the effort to incorporate research evidence into my practice. |  |
| Cognitive interviews | Changed the focus on the idea of “worth” to the concept of “requiring effort” to decrease social desirability bias. | | | |
| Version 8 | It requires **little effort** to integrate research evidence into practice. | It requires **some effort** to integrate research evidence into practice. | It requires **a lot of effort** to integrate research evidence into practice. |  |
| Cognitive interviews | Changed "some" effort to "moderate" effort. | | | |
| Version 9 | It requires **little effort** to integrate research evidence into practice. | It requires **moderate effort** to integrate research evidence into practice. | It requires **a lot of effort** to integrate research evidence into practice. |  |
| Version 10 | Idem |  |  |  |
| Cognitive interviews | Added “(effort) for me”. | | | |
| Version 11 | It requires **little effort** for me to integrate research evidence into practice. | It requires **moderate effort** for me to integrate research evidence into practice. | It requires **a lot of effort** for me to integrate research evidence into practice. |  |
| Version 12 | Idem |  |  |  |
| Item: Activities related to EBP - Keep up to date | | | |  |
| Version 1 | In the past month, how often have you: made time to read research? | 0 (never) / 1 (monthly or less) / 2 (bi-weekly) / 3 (weekly). /4 (daily) |  |  |
| Focus group | Changed to declarative statements. Omitted the idea of making time to read research. Changed “reading research” to “consulting research evidence” to be more inclusive to other sources of research evidence. | | | |
| Version 2 | I regularly consult research evidence. | I occasionally consult research evidence. | I rarely consult research evidence. |  |
| Version 3 | Idem |  |  |  |
| Version 4 | Idem |  |  |  |
| Version 5 | Idem |  |  |  |
| Version 6 | Idem |  |  |  |
| Version 7 | Idem |  |  |  |
| Version 8 | Idem |  |  |  |
| Version 9 | Idem |  |  |  |
| Cognitive interviews | Changed "consult research evidence" to "keep up to date with research evidence" because participants interpreted the item as “frequency of using evidence in their practice” and the activity of keeping up with knowledge of scientific research outside of their clinical encounters. | | | |
| Version 10 | I **regularly** keep up to date with research evidence. | I **occasionally** keep up to date with research evidence. | I **rarely** keep up to date with research evidence. |  |
| Version 11 | Idem |  |  |  |
| Version 12 | Idem |  |  |  |
| Iterations of the instructions and changes to the visual presentation (in italics) | | | | |
| Version 1 | None |  |  |  |
| Version 2 | None |  |  |  |
| Research team | Developed and added instructions for the index. | | | |
| Version 3 | For each group of statements, select ONE statement which best applies to you. Please respond as honestly as possible. | | | |
| Cognitive interviews | Removed the last sentence from the previous version. Specified “best reflects your current practice” to contextualize the answers. Added the word “instructions”.  *Bolded the response options for each item.* | | | |
| Version 4 | Instructions: For each group of statements, please select ONE statement which best reflects your current practice. | | | |
| Cognitive interviews | Added “(best reflects your current practice) and context”. | | | |
| Version 5 | Instructions: For each group of statements, please select ONE statement which best reflects your current practice and context. | | | |
| Cognitive interviews | Replaced “For each group of statements” with “from each box”. *Added boxes around each of the five items. Supplemented the checkboxes for each response choice with numbers and letters.* | | | |
| Version 6 | Please **select** **ONE** statement **from** **each box** which best reflects your current practice and context. | | | |
| Version 7 | Idem |  |  |  |
| Version 8 | Idem |  |  |  |
| Version 9 | Idem |  |  |  |
| Version 10 | Idem |  |  |  |
| Version 11 | Idem |  |  |  |
| Version 12 | Idem |  |  |  |

**French version of the index**

| Question : Utilisation autodéclarée des données probantes | | | |  |
| --- | --- | --- | --- | --- |
| Version 1 | Depuis un mois, à quelle fréquence avez-vous... | Décider d’un plan d’action approprié intégrant des données probantes, le jugement clinique et les préférences du client ou patient? | 0 (jamais) / 1 (une fois ou plus) |  |
| Groupe de discussion | Changed the initial item structure to three declarative statements. Changed "[integrating the three pillars]" to « *intégrer les évidences scientifiques »*. Changed « *décider d’un plan d’action approprié »* to « …*dans le plan de soins »*. | | | |
| Version 2 | J’intègre régulièrement les évidences scientifiques dans le plan de soins. | J’intègre occasionnellement les évidences scientifiques dans le plan de soins. | J’intègre rarement ou jamais les évidences scientifiques dans le plan de soins. |  |
| Équipe de recherche | Changed item to elicit the first source of information when faced with a practice uncertainty. | | | |
| Version 3 | Face à une incertitude dans ma pratique, je m'appuie sur les évidences scientifiques. | Face à une incertitude dans ma pratique, je m'appuie sur mes collègues. | Face à une incertitude dans ma pratique, je m'appuie sur mon expérience clinique. |  |
| Version 4 | Idem |  |  |  |
| Version 5 | Idem |  |  |  |
| Entretiens cognitifs | Added an asterisk to define « *incertitude dans ma pratique »*. | | | |
| Version 6 | Je m'appuie premièrement sur **les évidences scientifiques** lorsque je fais face à une incertitude* dans ma pratique. | Je m'appuie premièrement sur **mes collègues** lorsque je fais face à une incertitude* dans ma pratique. | Je m'appuie premièrement sur **mon expérience clinique** lorsque je fais face à une incertitude* dans ma pratique. | **incertitude : une situation dans laquelle il existe un déficit dans vos connaissances en lien avec une décision clinique* |
| Version 7 | Idem |  |  |  |
| Version 8 | Idem |  |  |  |
| Version 9 | Idem |  |  |  |
| Version 10 | Idem |  |  |  |
| Entretiens cognitifs | To decrease variability due to clinical population and context, the item was modified to focus on frequency of using research evidence when faced with a practice uncertainty. | | | |
| Version 11 | Lorsque je fais face à une incertitude* dans ma pratique,  j’utilise **presque toujours** les données probantes. | j’utilise **parfois** les données probantes. | j’utilise **rarement** les données probantes. | **incertitude : une situation dans laquelle il existe une lacune dans vos connaissances concernant une décision clinique.* |
| Version 12 | Idem |  |  |  |
| Question : Auto-efficacité | | | |  |
| Version 1 | Veuillez indiquer à quel point vous avez confiance en vos capacités actuelles en choisissant le nombre correspondant sur l’échelle d’appréciation suivante. | Déterminer si des preuves découlant d’une recherche de la littérature s’appliquent à la situation de votre patient ou client? | 0 (aucune confiance à 25%)) / 1 (25-50%) / 2 (50-75%) /3 (75% à confiance totale) |  |
| Groupe de discussion | Changed the initial item structure to three declarative statements. Omitted « *confiance en vos capacités actuelles »* to shorten the sentence. Changed « *preuves découlant d’une recherche de la littérature* *»* to be inclusive of other sources of research evidence. | | | |
| Version 2 | Je suis confiant.e de décider si les évidences scientifiques s’appliquent à mes patients*. | Je suis plutôt confiant.e de décider si les évidences scientifiques s’appliquent à mes patients*. | Je ne suis pas confiant.e de décider si les évidences scientifiques s’appliquent à mes patients*. | *Patient et client sont utilisés comme synonymes |
| Équipe de recherche | Changed « décider si les évidences scientifiques s’appliquent à mes patients *»* to « *d'intégrer les évidences scientifiques dans mon plan d'intervention »*. Added « très *»* to first and « peu *»* to last levels. | | | |
| Version 3 | Je suis très confiant(e) en mes capacités d'intégrer les évidences scientifiques dans mon plan d'intervention. | Je suis moyennement confiant(e) en mes capacités d'intégrer les évidences scientifiques dans mon plan d'intervention. | Je suis peu confiant(e) en mes capacités d'intégrer les évidences scientifiques dans mon plan d'intervention. |  |
| Version 4 | Idem |  |  |  |
| Entretiens cognitifs | Changed « *d'intégrer* *»* to « *d'appliquer* *»*. Changed « *plan d'intervention* *»* to *« cas cliniques* *»*. | | | |
| Version 5 | Je suis **très confiant(e)** en mes capacités d'appliquer les évidences scientifiques à des cas cliniques. | Je suis **moyennement confiant(e)** en mes capacités d'appliquer les évidences scientifiques à des cas cliniques. | Je suis **peu confiant(e)** en mes capacités d'appliquer les évidences scientifiques à des cas cliniques. |  |
| Version 6 | Idem |  |  |  |
| Version 7 | Idem |  |  |  |
| Version 8 | Idem |  |  |  |
| Entretiens cognitifs | Changed « *très confiant(e) »* to « *confiant(e) ».* | | | |
| Version 9 | Je suis **confiant(e)** en mes capacités d'appliquer les évidences scientifiques à des cas cliniques. | Je suis **moyennement confiant(e)** en mes capacités d'appliquer les évidences scientifiques à des cas cliniques. | Je suis **peu confiant(e)** en mes capacités d'appliquer les évidences scientifiques à des cas cliniques. |  |
| Entretiens cognitifs | Changed *« cas cliniques* *»* to *« en pratique* *»*. | | | |
| Version 10 | Je suis **confiant(e)** en mes capacités d'appliquer les données probantes en pratique. | Je suis **moyennement confiant(e)** en mes capacités d'appliquer les données probantes en pratique. | Je suis **peu confiant(e)** en mes capacités d'appliquer les données probantes en pratique. |  |
| Version 11 | Idem |  |  |  |
| Version 12 | Idem |  |  |  |
| Question : Ressources | | | |  |
| Version 1 | Veuillez indiquer à quel point vous êtes en accord avec les énoncés suivants. | Mon organisation soutient les pratiques optimales. | 0 (tout à fait en désaccord /en désaccord)) / 1 (neutre) /2 (d’accord) /3 (tout à fait d’accord) |  |
| Groupe de discussion | Changed the initial item structure to three declarative statements. Changed *«* *pratiques optimales »* to *«* *la pratique basée sur les données probantes ».* | | | |
| Version 2 | Mon [organisation/milieu de clinique] soutient la pratique basée sur les données probantes. | Mon [organisation/milieu de clinique] soutient plus ou moins la pratique basée sur les données probantes. | Mon [organisation/milieu de clinique] ne soutient pas la pratique basée sur les données probantes. |  |
| Équipe de recherche | Changed *« Mon [organisation/milieu de clinique] soutient* …*»*  to *«* *J'estime avoir les ressources nécessaires* …*».* | | | |
| Version 3 | J'estime avoir les ressources nécessaires pour intégrer les évidences scientifiques dans ma pratique. | J'estime avoir seulement une partie des ressources nécessaires pour intégrer les évidences scientifiques dans ma pratique. | J'estime ne pas avoir les ressources nécessaires pour intégrer les évidences scientifiques dans ma pratique. |  |
| Version 4 | Idem |  |  |  |
| Entretiens cognitifs | Omitted *«* *J'estime (avoir)* *»*. Changed *«* *ne pas avoir les ressources*…*»* to *«* *(avoir) peu de ressources nécessaires»*. Added an asterisk with examples of resources. | | | |
| Version 5 | J'ai **les ressources nécessaires** pour intégrer les évidences scientifiques dans ma pratique. | J'ai **seulement une partie des ressources nécessaires** pour intégrer les évidences scientifiques dans ma pratique. | J'ai **peu de ressources nécessaires** pour intégrer les évidences scientifiques dans ma pratique. | **Exemples : du temps payé pour lire les données probantes, accès aux journaux, accès au matériel thérapeutique nécessaire* |
| Version 6 | Je possède **les ressources* nécessaires** pour intégrer les données probantes dans ma pratique. | Je possède **une partie des ressources* nécessaires** pour intégrer les données probantes dans ma pratique. | Je possède **peu de ressources* nécessaires** pour intégrer les données probantes dans ma pratique. | **Exemples : du temps payé pour lire les données probantes, accès aux journaux, accès au matériel thérapeutique nécessaire* |
| Entretiens cognitifs | Added computer to the examples. | | | |
| Version 7 | Idem |  |  | **Exemples : du temps payé pour lire les données probantes, accès aux journaux, accès à un ordinateur, accès au matériel thérapeutique nécessaire* |
| Version 8 | Idem |  |  |  |
| Version 9 | Idem |  |  |  |
| Version 10 | Idem |  |  |  |
| Version 11 | Idem |  |  |  |
| Version 12 | Idem |  |  |  |
| Question: Attitudes - Effort | | | |  |
| Version 1 | Veuillez indiquer à quel point vous êtes en accord avec les énoncés suivants. | J’accepterais de bon gré d’utiliser divers types d’interventions cliniques inédites (ex. évaluation, traitement) mises au point par des chercheurs pour aider mes patients ou clients. | 0 (tout à fait en désaccord /1 (en désaccord)) / 2 (neutre) /3 (d’accord) /4 (tout à fait d’accord) |  |
| Groupe de discussion | Changed the initial item structure to three declarative statements. Replaced the ending « *pour aider mes patients ou clients* » with *« pour le plan de soins de mes patients* ». Replaced «*d’utiliser divers types d’interventions cliniques inédites (ex. évaluation, traitement) mises au point par des chercheurs* » with « *utiliser des données probantes* » to simplify the item. | | | |
| Version 2 | Je suis [prêt à/partant de/tenté de] utiliser des données probantes pour le plan de soins de mes patients*. | Je suis plus ou moins [prêt à/partant de/tenté de] utiliser des données probantes pour le plan de soins de mes patients*. | Je ne suis pas [prêt à/partant de/tenté de] utiliser des données probantes pour le plan de soins de mes patients*. |  |
| Équipe de recherche | Changed « *Je suis prêt à utiliser des données probantes*…» to *« en valloir la peine* » to diminish social desirability bias. | | | |
| Version 3 | Intégrer les évidences scientifiques dans ma pratique en vaut vraiment la peine. | Intégrer les évidences scientifiques dans ma pratique en vaut plus ou moins la peine. | Intégrer les évidences scientifiques dans ma pratique n'en vaut pas la peine. |  |
| Version 4 | Idem |  |  |  |
| Entretiens cognitifs | Inversed the structure of the sentence. Added « *n'en vaut pas vraiment la peine* ». Added «… *la peine pour moi* »*.* | | | |
| Version 5 | Cela **vaut** **vraiment** **la peine** pour moi d'intégrer les évidences scientifiques dans ma pratique. | Cela **vaut plus ou moins** **la peine** pour moi d'intégrer les évidences scientifiques dans ma pratique. | Cela **n'en vaut pas vraiment la peine** pour moi d'intégrer les évidences scientifiques dans ma pratique. |  |
| Version 6 | Idem |  |  |  |
| Entretiens cognitifs | Changed « (valloir) *la peine pour moi* » to « (valloir) *l’effort* ». Changed the focus on the idea of “worth” to the concept of “requiring effort” to decrease social desirability bias. Removed the superlative « *vraiment* » from first response option. | | | |
| Version 7 | Cela **vaut l'effort** d'intégrer les évidences scientifiques dans ma pratique. | Cela **vaut moyennement** **l'effort** d'intégrer les évidences scientifiques dans ma pratique. | Cela **ne vaut pas vraiment** **l'effort** d'intégrer les évidences scientifiques dans ma pratique. |  |
| Entretiens cognitifs | Replaced « (valloir) l’effort » with « nécessite de l’effort ». | | | |
| Version 8 | Cela **nécessite peu d'efforts** pour intégrer les évidences scientifiques dans ma pratique. | Cela **nécessite quelques efforts** pour intégrer les évidences scientifiques dans ma pratique. | Cela **nécessite beaucoup d'efforts** pour intégrer les évidences scientifiques dans ma pratique. |  |
| Entretiens cognitifs | Changed « *quelques efforts* » to « *effort modéré* ». | | | |
| Version 9 | Cela **nécessite** **peu d'effort** pour intégrer les évidences scientifiques dans ma pratique. | Cela **nécessite** **un effort modéré** pour intégrer les évidences scientifiques dans ma pratique. | Cela **nécessite** **beaucoup d'effort** pour intégrer les évidences scientifiques dans ma pratique. |  |
| Version 10 | Idem |  |  |  |
| Entretiens cognitifs | Starting the sentence with “cela” was problematic in French; sentence structure was inversed from English. Added “*me (demande)*” to specify that the focus is on the respondent’s perceived effort. | | | |
| Version 11 | Intégrer les données probantes dans ma pratique me demande **peu d'effort.** | Intégrer les données probantes dans ma pratique me demande **un effort modéré**. | Intégrer les données probantes dans ma pratique me demande **beaucoup d'effort**. |  |
| Version 12 | Idem |  |  |  |
| Question : Activités reliées à la PFDP - Se tenir à jour | | | |  |
| Version 1 | Depuis un mois, à quelle fréquence avez-vous... | Réservé du temps à la lecture de travaux de recherche? | 0 (jamais) / 1 (une fois par mois ou moins) / 2 (aux 2 semaines) / 3 (toutes les semaines) / 4 (tous les jours) |  |
| Groupe de discussion | Changed to declarative statements. Omitted the idea of making time to read research. Changed « *la lecture de travaux de recherche* » to « *consulter les évidences scientifiques* » to be more inclusive of other sources of research evidence. | | | |
| Version 2 | Je consulte régulièrement les évidences scientifiques. | Je consulte occasionnellement les évidences scientifiques. | Je consulte rarement ou jamais les évidences scientifiques. |  |
| Version 3 | Idem |  |  |  |
| Version 4 | Idem |  |  |  |
| Version 5 | Idem |  |  |  |
| Version 6 | Idem |  |  |  |
| Version 7 | Idem |  |  |  |
| Version 8 | Idem |  |  |  |
| Version 9 | Idem |  |  |  |
| Entretiens cognitifs | Changed « *consulter les évidences scientifiques* » to « *(se tenir) à jour quant aux données probantes* » because participants interpreted the item as “frequency of using evidence in their practice” and the activity of keeping up with knowledge of scientific research outside of their clinical encounters. | | | |
| Version 10 | Je me tiens **régulièrement** à jour quant aux données probantes. | Je me tiens **occasionnellement** à jour quant aux données probantes. | Je me tiens **rarement** à jour quant aux données probantes. |  |
| Version 11 | Idem |  |  |  |
| Version 12 | Idem |  |  |  |
| Iterations of the instructions and changes to the visual presentation (in italics) | | | | |
| Version 1 | None |  |  |  |
| Version 2 | None |  |  |  |
| Équipe de recherche | Developed and added instructions for the index. | | | |
| Version 3 | Pour chaque groupe d'énoncés, veuillez sélectionner UN énoncé qui s'applique le mieux à vous. Merci de répondre le plus honnêtement possible. | | | |
| Entretiens cognitifs | Removed the last sentence from the previous version. Specified *« (s'applique le mieux à) votre pratique* » to contextualize the answers. Added the word “*instructions*”. *Bolded the response options for each item.* | | | |
| Version 4 | Instructions: Pour chaque groupe d'énoncés, veuillez sélectionner UN énoncé qui reflète le mieux votre pratique. | | | |
| Entretiens cognitifs | Added « *(s'applique le mieux à) votre pratique et contexte actuel* ». | | | |
| Version 5 | Instructions: Pour chaque groupe d'énoncés, veuillez sélectionner UN énoncé qui reflète le mieux votre pratique et contexte actuel. | | | |
| Entretiens cognitifs | Replaced « *Pour chaque groupe d'énoncés* » with « *de chaque case* ». *Added boxes around each of the five items. Supplemented the checkboxes for each response choice with numbers and letters. Bolded key words in the instructions.* | | | |
| Version 6 | Veuillez **sélectionner UN** énoncé **de chaque case** qui reflète le mieux votre pratique et contexte actuel. | | | |
| Version 7 | Idem |  |  |  |
| Version 8 | Idem |  |  |  |
| Version 9 | Idem |  |  |  |
| Version 10 | Idem |  |  |  |
| Version 11 | Idem |  |  |  |
| Version 12 | Idem |  |  |  |

### Appendix III. Overview of the item evolution process during cognitive interviews

| Participant # | Language | Profession | Index version | Use of RE | Self-efficacy | Resources | Attitudes | Activities related to EBP | Instructions | Visual |
| --- | --- | --- | --- | --- | --- | --- | --- | --- | --- | --- |
| P1 | EN | PT | 3 | Major | Minor | Major | Minor |  | Minor | Minor |
| P2 | EN | PT | 3 | Minor |  |  | Minor |  | Minor |  |
| P3 | EN | OT | 3 | Major |  | Minor | Minor |  | Minor |  |
| P4 | FR | OT | 3 | Minor | Major | Minor | Minor |  | Minor |  |
| *New version 4* | | | | **M** |  | **M** | **M** |  | **M** | **M** |
| P5 | EN | OT | 4 |  | Minor | Minor |  |  |  |  |
| *New version 5* | | | |  | **M** | **M** | **M** |  | **M** |  |
| P6 | FR | OT | 5 | Minor |  | Minor |  |  |  |  |
| P7 | EN | OT | 5 | Minor |  |  |  |  | Minor | Minor |
| P8 | EN | PT | 5 | Minor | Minor |  | Major |  | Minor |  |
| *New version 6* | | | | **M** |  |  |  |  | **M** | **M** |
| P9 | FR | PT | 6 |  |  |  | Minor | Minor |  |  |
| P10 | FR | OT | 6 |  |  | Minor | Minor |  |  |  |
| *New version 7* | | | |  |  | **M** | **M** |  |  |  |
| P11 | FR | PT | 7 |  | Minor |  | Major |  |  |  |
| P12 | FR/EN | OT | 7 |  | Minor | Minor | Minor |  |  |  |
| P13 | FR | PT | 7 | Minor |  |  | Major |  |  |  |
| *New version 8* | | | |  |  |  | **M** |  |  |  |
| P14 | EN | PT | 8 | Minor |  |  | Minor |  |  |  |
| P15 | EN | OT | 8 |  | Minor |  | Minor |  |  |  |
| *New version 9* | | | |  | **M** |  | **M** |  |  |  |
| P16 | EN | OT | 9 |  | Minor |  |  | Minor |  |  |
| P17 | FR | PT | 9 |  |  |  | Minor | Minor |  |  |
| *New version 10* | | | |  | **M** |  |  | **M** |  |  |
| P18 | FR | OT | 10 |  |  |  |  |  |  |  |
| P19 | FR | OT | 10 | Major |  |  | Minor | Minor |  |  |
| P20 | FR | OT | 10 | Minor | Minor | Minor |  |  |  |  |
| P21 | EN | OT | 10 | Minor |  |  |  |  |  |  |
| *New version 11* | | | | **M** |  |  | **M** |  |  |  |
| P22 | FR | PT | 11 |  |  |  |  |  |  |  |
| P23 | EN | OT | 11 |  |  |  |  |  |  |  |
| P24 | EN | PT | 11 |  |  |  |  | Minor |  |  |
| *Version 12 accepted* | | | |  |  |  |  |  |  |  |
| Total iterations during cognitive interviews | | | | 3 | 3 | 3 | 6 | 1 |  |  |
| EN: English; FR: French; PT: physical therapist; OT: occupational therapist; RE: research evidence; Major: Major issue identified by participants; Minor: minor issue identified by participants; M: Modification implemented by the research team into next version of the index | | | | | | | | | | |

Appendix IV. Final index in English and French

**Propensity to Integrate Research Evidence into Clinical Decision-Making Index (PIRE-CDMI)**

Please **select ONE** statement **from each box** which best reflects your current practice and context.

1. When faced with a practice uncertainty*,

| ☐ | a) I **almost always** use research evidence. |
| --- | --- |
| ☐ | b) I **sometimes** use research evidence. |
| ☐ | c) I **rarely** use research evidence. |
|  | **uncertainty: a situation in which there is a gap in your knowledge relating to a clinical decision* |

2.

| ☐ | a) I am **confident** in my ability to apply research evidence to practice. |
| --- | --- |
| ☐ | b) I am **somewhat confident** in my ability to apply research evidence to practice. |
| ☐ | c) I am **not very confident** in my ability to apply research evidence to practice. |

3.

| ☐ | a) I have **the necessary resources*** to integrate research evidence into my practice. |
| --- | --- |
| ☐ | b) I have **some of the necessary resources*** to integrate research evidence into my practice. |
| ☐ | c) I have **few of the necessary resources*** to integrate research evidence into my practice. |
|  | **Examples: paid time to consult the evidence, access to journals, access to a computer, access to necessary therapeutic material...* |

4.

| ☐ | a) It requires **little effort** for me to integrate research evidence into practice. |
| --- | --- |
| ☐ | b) It requires **moderate effort** for me to integrate research evidence into practice. |
| ☐ | c) It requires **a lot of effort** for me to integrate research evidence into practice. |

5.

| ☐ | a) I **regularly** keep up to date with research evidence. |
| --- | --- |
| ☐ | b) I **occasionally** keep up to date with research evidence. |
| ☐ | c) I **rarely** keep up to date with research evidence. |

**Indice de la tendance à intégrer les données probantes dans la prise de décisions cliniques (I-TIDP-PDC)**

Veuillez **sélectionner UN** énoncé **de chaque encadré** qui reflète le mieux votre pratique et contexte actuel.

1. Lorsque je fais face à une incertitude* dans ma pratique,

| ☐ | a) J’utilise **presque toujours** les données probantes. |
| --- | --- |
| ☐ | b) J’utilise **parfois** les données probantes. |
| ☐ | c) J’utilise **rarement** les données probantes. |
|  | **incertitude: une situation dans laquelle il existe une lacune dans vos connaissances concernant une décision clinique.* |

2.

| ☐ | a) Je suis **confiant(e)** en mes capacités d'appliquer les données probantes en pratique. |
| --- | --- |
| ☐ | b) Je suis **moyennement confiant(e)** en mes capacités d'appliquer les données probantes en pratique. |
| ☐ | c) Je suis **peu confiant(e)** en mes capacités d'appliquer les données probantes en pratique. |

3.

| ☐ | a) Je possède **les ressources* nécessaires** pour intégrer les données probantes dans ma pratique. |
| --- | --- |
| ☐ | b) Je possède **une partie des ressources* nécessaires** pour intégrer les données probantes dans ma pratique. |
| ☐ | c) Je possède **peu de ressources* nécessaires** pour intégrer les données probantes dans ma pratique. |
|  | **Exemples : du temps payé pour lire la littérature, accès aux journaux, accès à un ordinateur, accès au matériel thérapeutique nécessaire* |

4.

| ☐ | a) Intégrer les données probantes dans ma pratique me demande **peu d'effort**. |
| --- | --- |
| ☐ | b) Intégrer les données probantes dans ma pratique me demande un **effort modéré**. |
| ☐ | c) Intégrer les données probantes dans ma pratique me demande **beaucoup d'effort**. |

5.

| ☐ | a) Je me tiens **régulièrement** à jour quant aux données probantes. |
| --- | --- |
| ☐ | b) Je me tiens **occasionnellement** à jour quant aux données probantes. |
| ☐ | c) Je me tiens **rarement** à jour quant aux données probantes. |
